# Use of Methylene Blue in Patients With Shock in the Intensive Care Unit Over a 10-Year Period at a Private Hospital in Costa Rica

**DOI:** 10.7759/cureus.94124

**Published:** 2025-10-08

**Authors:** Esteban Zavaleta-Monestel, Jimena Campos, Abigail Fallas-Mora, Sebastián Arguedas-Chacón, José Chaverri-Fernández

**Affiliations:** 1 Pharmacy, Hospital Clinica Biblica, San José, CRI; 2 Department of Pharmacy, University of Costa Rica, San José, CRI; 3 Department of Pharmacology, Toxicology and Drug Dependence, University of Costa Rica, San José, CRI; 4 Pharmacology and Therapeutics, Hospital Clínica Bíblica, San José, CRI; 5 Research, Hospital Clinica Biblica, San José, CRI

**Keywords:** intensive care unit, methylene blue, retrospective study, shock, vasopressor agents

## Abstract

Introduction

Methylene blue (MB) has been proposed as an adjunctive therapy for the management of refractory shock; however, clinical evidence from Latin America remains limited. This study evaluated its impact on hemodynamic parameters and clinical outcomes in critically ill patients. Responders were defined as those who demonstrated hemodynamic improvement after MB administration, meeting at least one of the following criteria: an increase of ≥10% in mean arterial pressure (MAP), a reduction of ≥10% in vasoactive-inotropic score (VIS), or a ≥10% decrease in serum lactate levels.

Methods

A retrospective cohort study was conducted in the intensive care unit (ICU) of Hospital Clínica Bíblica, a private hospital in Costa Rica, between January 2014 and December 2024. A total of 98 adult patients with refractory shock who received MB were included. Clinical and laboratory variables were assessed before and after treatment. Response was defined as a composite endpoint (≥10% improvement in MAP, ≥10% reduction in VIS, or ≥10% decrease in lactate). Statistical analysis included Wilcoxon signed-rank tests, ANOVA/Kruskal-Wallis, and logistic regression models.

Results

The mean age was 72.6 ± 14.1 years, and 65.3% were male. Septic shock was the most frequent etiology (55.1%). Thirty-day survival did not differ significantly across shock types (log-rank p = 0.171), although clinically relevant trends were observed. Overall, 73.5% (n = 72) of patients met the composite response criteria. Significant improvements were documented in the MAP (70.5 ± 19.3 to 74.2 ± 21.9 mmHg; p < 0.001) and shock index (p = 0.003). VIS increased at the population level (median 6.0-21.0; p < 0.001), reflecting heterogeneity in response. Bolus or bolus plus infusion strategies showed higher response rates than continuous infusion (82.9%, 90.0%, and 62.3%, respectively; p = 0.045). Lower pre-treatment VIS was independently associated with higher odds of response (aOR = 0.979, p = 0.016), and higher post-treatment MAP was associated with reduced mortality risk (aOR = 0.933, p = 0.005).

Conclusions

MB may represent a beneficial therapeutic option in selected patients with refractory shock. Bolus administration, alone or combined with infusion, appeared more effective than continuous infusion. MAP and VIS may serve as dynamic markers of response, although VIS was heterogeneous and influenced by outliers. Prospective, multicenter studies are required to confirm these findings and identify patient subgroups most likely to benefit.

## Introduction

Shock is a life-threatening condition associated with high morbidity and mortality in intensive care unit (ICU) patients. It is commonly classified into cardiogenic, hypovolemic, obstructive, and distributive types, with the latter being the most prevalent [1]. Refractory shock represents a major clinical challenge due to its resistance to conventional therapy with fluids and vasopressors. It is characterized by organ dysfunction caused by an imbalance between oxygen delivery and consumption at the cellular level, leading to tissue hypoxia. This imbalance may result from loss of vascular tone or impaired cardiac output. Microcirculatory alterations trigger immune activation and the release of proinflammatory mediators such as tumor necrosis factor-alpha (TNF-α) and interleukin-6 (IL-6), which induce the expression of inducible nitric oxide synthase (iNOS) and excessive nitric oxide (NO) production. NO acts as a potent vasodilator, reducing vascular tone, mean arterial pressure (MAP), and organ perfusion. Microcirculatory dysfunction and vasoplegia are therefore key mechanisms in the progression of refractory shock [2].

Early management aims to prevent irreversible organ failure through intravenous fluid resuscitation and vasopressor support, primarily with norepinephrine, to achieve an MAP ≥65 mmHg. However, in some patients, conventional therapy remains insufficient even at high vasopressor doses, exacerbating hemodynamic instability and increasing mortality. Thus, the choice, timing, and combination of vasoactive agents may significantly influence outcomes [3].

Methylene blue (MB) has been proposed as an adjunctive therapy to restore vascular tone by inhibiting endothelial (eNOS) and inducible (iNOS) nitric oxide synthase, as well as soluble guanylate cyclase (sGC), thereby reducing cyclic guanosine monophosphate (cGMP) levels and counteracting vasoplegia [4-6]. MB has been reported to decrease catecholamine requirements, increase MAP, and improve systemic vascular resistance [7]. Its clinical effect peaks within 30 minutes of intravenous administration, with a half-life of six to 24 hours. Because of its large volume of distribution (255 L) and high protein binding (94%), the pharmacodynamic response can vary. Administration strategies include bolus, continuous infusion, or a combination of both. Evidence suggests that early administration may enhance hemodynamic stability and reduce vasopressor dependency; however, its efficacy remains uncertain, particularly in low- and middle-income settings, including Latin America [8].

Despite its reported use, clinical evidence on the impact of MB on hemodynamic parameters and outcomes remains limited and heterogeneous, and standardized response criteria are lacking. Therefore, generating regional data is essential to characterize treated populations, describe their clinical course, and identify predictors of therapeutic response.

This study aims to evaluate the effect of MB on hemodynamic stability and clinical outcomes in patients with refractory shock treated in the ICU of a private tertiary hospital in Costa Rica. Although this is a single-center study, the ICU provides cardiovascular and vasopressor management comparable to international standards, making it a relevant setting for regional evidence generation. Secondary objectives include describing patient characteristics, treatment strategies, and potential predictors of clinical response and mortality.

## Materials and methods

Study population and ethics

An observational, retrospective, and analytical study was conducted through the review of medical records of patients admitted to the intensive care unit (ICU) of Hospital Clínica Bíblica, Costa Rica, who received MB between January 2014 and December 2024. Due to its retrospective nature, no sample size calculation was performed, and all patients meeting the inclusion criteria were analyzed. 

Patients aged 18 years or older admitted to the ICU with a diagnosis of refractory shock, defined as persistent hypotension (MAP <65 mmHg) despite adequate fluid resuscitation and high-dose vasopressor therapy, typically norepinephrine ≥0.3 µg/kg/min or equivalent for at least 30 minutes [1-2], of septic, cardiogenic, hypovolemic, distributive, or mixed origin, and who received MB as part of their management, were included. Patients were excluded if their medical records were incomplete, lacked sufficient clinical data, or had absolute contraindications to MB (e.g., glucose-6-phosphate dehydrogenase deficiency, known hypersensitivity, concomitant use of selective serotonin reuptake inhibitors, and pregnancy), as well as those with shock secondary to poisoning or other non-vasoplegic causes. Patient classification was based on information documented in ICU clinical records. Of the 113 patients initially identified, 15 were excluded due to incomplete data, resulting in a final sample of 98 cases for analysis.

This study was approved by the Scientific Ethics Committee of the University of Costa Rica (CEC-UCR) under protocol number CEC-346-2025. Given its retrospective and observational design, as well as the use of anonymized data, individual informed consent was not required. All data were retrospectively collected and anonymized prior to analysis, in compliance with ethical standards and local regulations. 

Data collection

Data were collected retrospectively through manual review of institutional electronic medical records (SIGH) and the Hospital Process Manager (GPH). Collected variables included demographic characteristics, admission diagnosis, clinical outcomes, comorbidities, type and dosage of vasopressors, use of other medications, hemodynamic parameters (MAP, heart rate (HR), respiratory rate (RR)), and laboratory values (lactate, pH, pCO₂, bicarbonate (HCO₃⁻), blood urea nitrogen (BUN), creatinine (Cr)). These variables were recorded at two time points: before and after MB administration. Data were entered into an anonymized spreadsheet by a single investigator and verified by a second reviewer to ensure consistency. 

Vasoactive-Inotropic Score (VIS)

Patients received different vasoactive agents, and some were treated with multiple agents simultaneously (inotropes such as dobutamine and vasopressors such as norepinephrine). Vasoactive agents were typically used concurrently rather than sequentially. The VIS was calculated based on the total simultaneous infusion rates at the time of measurement. The Vasoactive-Inotropic Score (VIS) was used to quantify the amount of hemodynamic support required by each patient. This index was calculated using the formula described by Gaies et al., which incorporates the weighted doses of multiple inotropic and vasopressor agents. The VIS has been validated by multicenter and cohort studies for use in critically ill adults as a composite marker of vasoactive load and a predictor of clinical outcomes in the ICU [9-11]. The score integrates the weighted doses of multiple inotropes and vasopressors as follows:

VIS = dopamine (µg/kg/min) + dobutamine (µg/kg/min) + [100 × epinephrine] + [100 × norepinephrine] + [10,000 × vasopressin] + [10 × milrinone]

Clinical response

Clinical response to MB was evaluated based on comorbidities (Charlson Comorbidity Index, CCI), shock index (SI), VIS, and pre- and post-treatment hemodynamic and laboratory parameters [12,13]. Responders were defined as patients who demonstrated hemodynamic improvement following administration, based on meeting at least one of the following criteria: an increase of ≥10% in the MAP, a reduction of ≥10% in VIS, or a ≥10% decrease in serum lactate levels. These thresholds were chosen based on prior literature and represent clinically meaningful changes in patients with refractory shock. 

Statistical analysis

Statistical analysis was performed using JASP software (version 0.19.3.0). The normality of continuous variables was assessed with the Shapiro-Wilk test and homoscedasticity with Levene’s test. Pre- and post-treatment comparisons were carried out using the Wilcoxon signed-rank test for paired samples. 

Between-group comparisons for continuous variables (e.g., according to MB administration strategy) were performed with one-way ANOVA when assumptions of normality and homoscedasticity were met or with the Kruskal-Wallis test otherwise. Categorical variables were compared using Pearson’s χ² or Fisher’s exact test, as appropriate. Survival analyses were conducted using Kaplan-Meier curves with log-rank testing. 

A multivariate binary logistic regression model was developed to assess independent predictors of clinical outcomes, using the “Enter” method. Variables were selected a priori based on clinical relevance and included post-treatment physiological parameters such as MAP, VIS, creatinine clearance (ClCr), CCI, and clinical response. Odds ratios (ORs), 95% confidence intervals (95% CI), and p-values were reported using the Wald test. 

To evaluate differences between responders and non-responders, Fisher’s exact test was used for categorical variables and the Mann-Whitney U test for continuous variables. Patients were classified as responders if they met at least one predefined response criterion. 

Results are presented as mean ± standard deviation (SD) when normally distributed, or as median and interquartile range (IQR) for skewed variables (e.g., VIS). Statistical significance was defined as p < 0.05 (two-sided). 

## Results

Patient selection and characteristics

**Figure 1:**
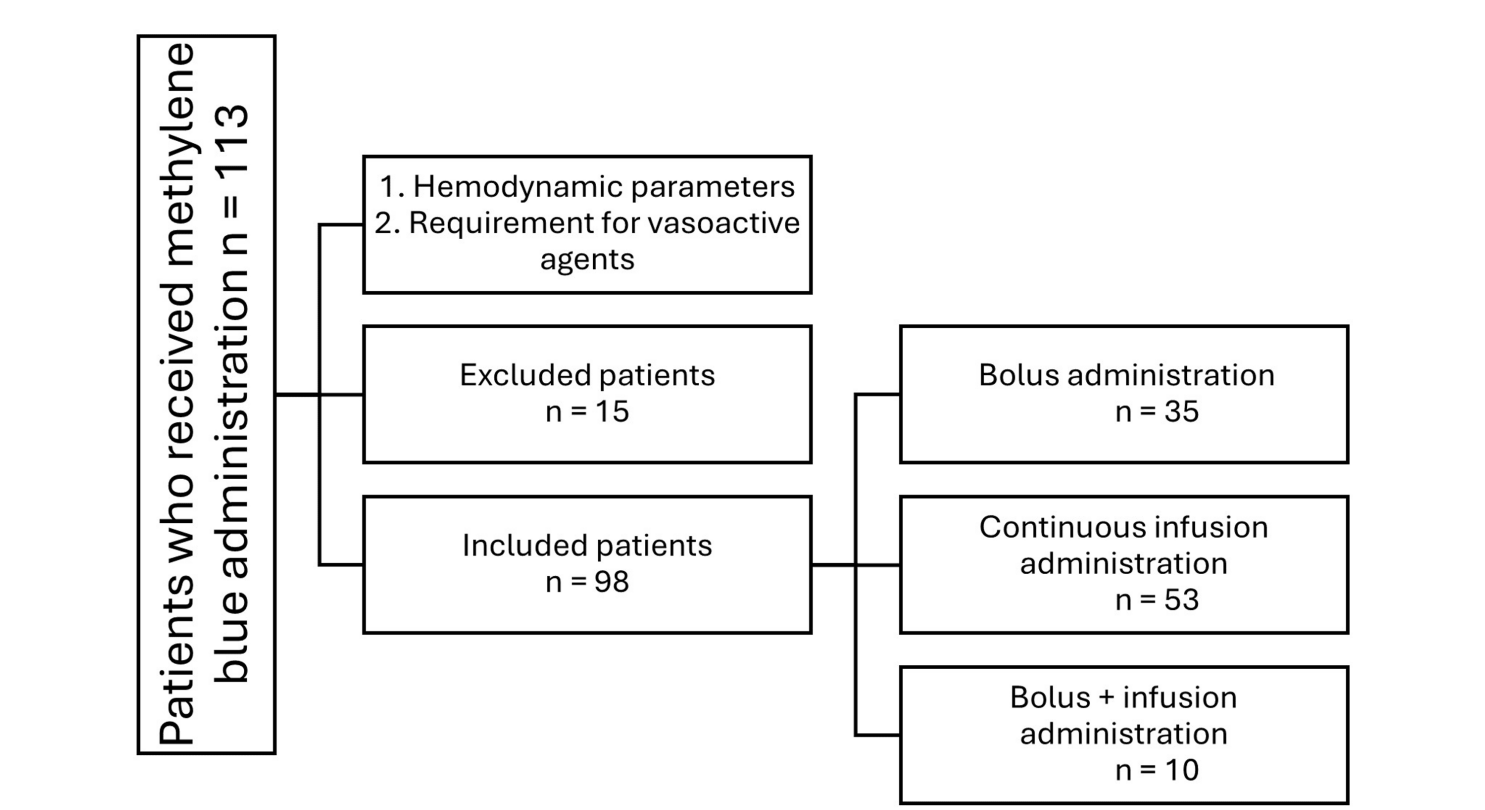
Patient selection process for the retrospective analysis. This figure illustrates the inclusion and exclusion criteria applied to identify the final cohort.

Figure 1 illustrates the selection process, identifying 98 eligible patients for this retrospective study.

As shown in Table 1, a total of 98 patients diagnosed with shock and treated with MB in the ICU were included. The mean age was 72.6 ± 14.1 years, with 65.3% (*n *= 64) being male. The average hospital stay was 13.4 days, and the ICU stay was 11.5 days. Regarding hospital outcomes, 76.5% (*n* = 75) died, 20.4% (*n *= 20) were transferred, and only 3.1% (*n* = 3) were discharged alive. Septic shock was the most common type (55.1%, *n *= 54), followed by distributive (15.3%, *n *= 15), cardiogenic (13.3%, *n *= 13), hypovolemic (10.2%, *n* = 10), and mixed shock (5.1%, *n *= 5). 

**Table 1:** Demographic characteristics of ICU patients with shock treated with methylene blue. Categorical variables are expressed as absolute frequencies and percentages (n, %), and continuous variables as mean ± standard deviation (SD). No comparative tests were applied. Statistical significance was defined as *p* < 0.05.

Variable		n = 98 (%)	Mean ± SD
Age	-	-	72.60 ± 14.16
Sex	Male	64 (65.3)	-
	Female	34 (37.7)	
Length of stay	Hospital	-	13.38 ± 16.31
	ICU	-	11.15 ± 15.15
Outcome	Deceased	75 (76.5)	-
	Transferred	20 (20.4)	
	Discharged	3 (3.1)	
Type of shock	Septic	54 (55.1)	-
	Distributive	15 (15.3)	
	Cardiogenic	13 (13.3)	
	Hypovolemic	10 (10.2)	
	Mixed	5 (5.1)	
	Unclassified	1 (1.0)	
Comorbidities	Cardiovascular	68 (69.4)	-
	Central Nervous System	51 (52.0)	
	Endocrine	35 (35.7)	
	Respiratory	27 (27.5)	
	Cancer	18 (18.4)	
	Other	18 (18.4)	
Charlson Comorbidity Index	Mild	5 (5.1)	-
	Moderate	25 (25.5)	
	Severe	49 (50.0)	
	Very Severe	19 (19.7)	
Route of administration	Bolus	35 (35.71)	-
	Infusion	53 (54.08)	
	Bolus + infusion	10 (10.20)	
Response	Responder	72 (73.47)	-
	Non-responder	26 (26.53)	-
30-day mortality	≥30 days	15 (15.31)	-
	<30 days	83 (84.69)	

Cardiovascular disease was the most prevalent comorbidity (69.4%, *n* = 68), followed by central nervous system disorders (52.0%,* n* = 51), endocrine conditions (35.7%, *n* = 35), and respiratory diseases (27.5%, *n* = 27). Fifty percent (*n* = 49) of the patients had a severe Charlson Comorbidity Index (CCI), and 19.7% (*n* = 19) had very severe comorbidity. Regarding administration strategy, MB was given via continuous infusion in 54.1% (*n *= 53), as a bolus in 35.7% (*n* = 35), and in bolus plus infusion combination in 10.2% (*n* = 10). A clinical response to MB was observed in 73.5% (*n* = 72) of patients. In addition, 15.3% (*n* = 15) of patients survived more than 30 days. 

As detailed in Table 2, during initial management, albumin was administered in 91.8% (*n* = 90) of cases, saline solution in 85.7% (*n* = 84), Ringer’s lactate in 64.3% (*n* = 63), and dextrose in 62.2% (*n* = 61). All patients (*n* = 98) received at least one type of intravenous fluid. Regarding vasoactive agents, the majority received norepinephrine (91.8%, *n* = 90), while 19.4% (*n *= 19) received dopamine, 8.2% (*n* = 8) epinephrine, and 22.4% (*n* = 22) dobutamine. 

**Table 2:** Frequency of fluid and vasopressor use during initial shock management. Values are expressed as *n* (%). No statistical comparisons were made. Statistical significance was defined as* p* < 0.05.

Type	Medications	n = 98 (%)
Fluids	Saline solution	84 (85.7)
	Dextrose	61 (62.2)
	Ringers lactate	63 (64.3)
	Albumin	90 (91.8)
Vasopresors	Norepinephrine	90 (91.8)
	Epinephrine	8 (8.2)
	Dopamine	19 (19.4)
Inotropes	Dobutamine	22 (22.4)

As presented in Table 3, etiologic analyses included 97 patients: septic (*n* = 54), non-septic distributive (*n* = 15), cardiogenic (*n *= 13), hypovolemic (*n* = 10), and mixed (*n* = 5). One patient with unclassified shock was excluded due to insufficient criteria for group assignment. The median survival was lowest in the cardiogenic group (two days), followed by the mixed (three days) and distributive (five days) groups. By contrast, the hypovolemic and septic groups showed a median survival of eight days. 

**Table 3:** Comparison of clinical parameters and outcomes by methylene blue administration strategy. This table presents the number of patients (*n*) categorized by shock subtype, the number and percentage of deaths occurring within 30 days, and the median survival time (in days) for each group. This information enables comparison of early mortality and clinical progression across different pathophysiological types of shock treated with methylene blue.

Type of shock	n	30-day mortality n(%)	Mean survival (days)
Cardiogenic	13	12 (92.31)	2
Distributive	15	11(73.33)	5
Hipovolemic	10	9 (90.00)	8
Mixed	5	5 (100.00)	3
Septic	54	45 (83.33)	8

**Figure 2:**
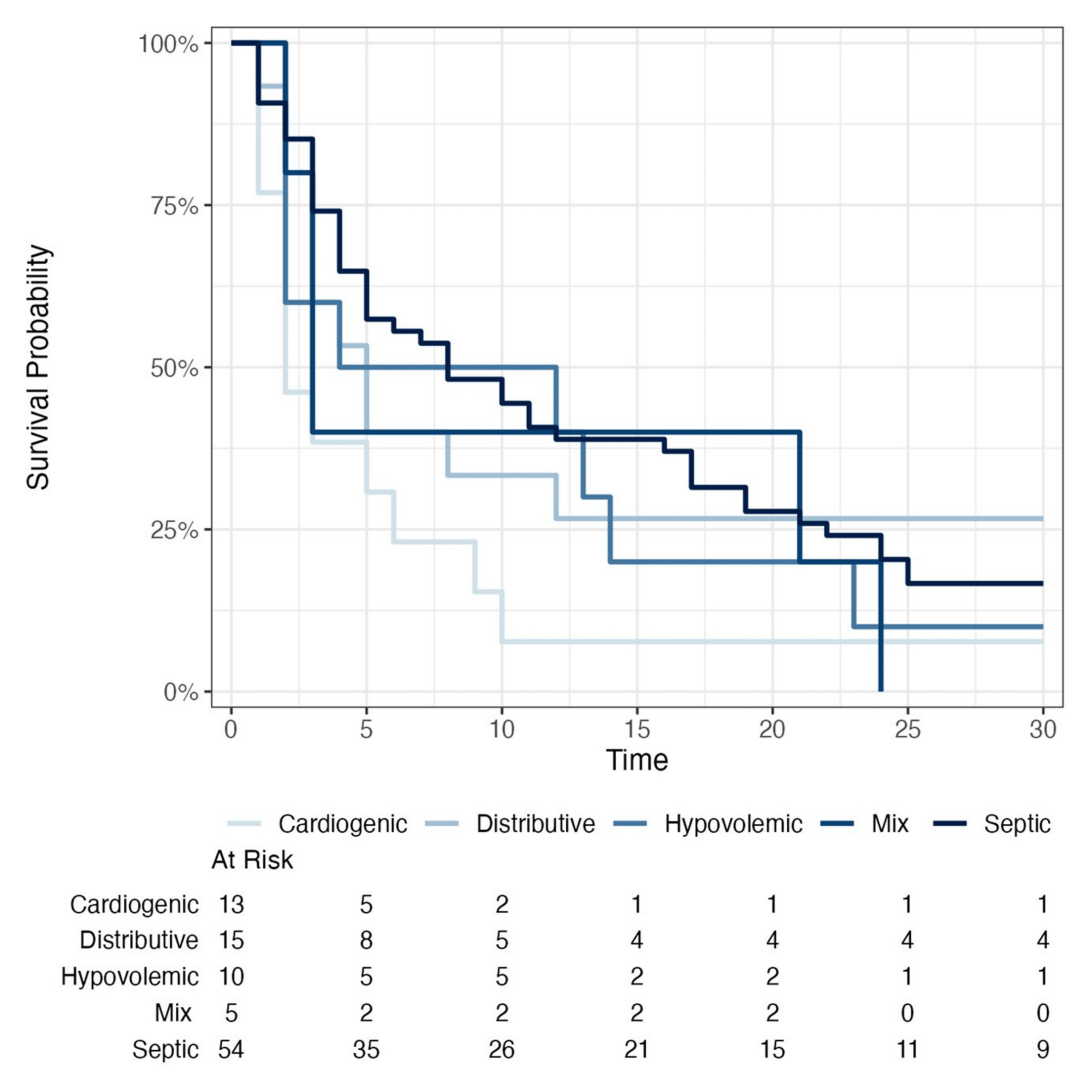
Kaplan-Meier survival curves over 30 days in patients with shock, stratified by shock type. Log-rank test: χ² = 6.398, df = 4, *p* = 0.171. Statistical significance was defined as *p* < 0.05.

The risk table illustrates the progression of each group throughout the follow-up period. In the cardiogenic shock group, only five out of 13 patients remained under observation by day 5, and just one by day 15, indicating a high early mortality. Patients with mixed shock experienced 100% mortality within 30 days. In the distributive shock group, eight patients remained at risk by day 5, and four were still under follow-up at day 30. By contrast, the septic shock group showed greater stability, with 35 patients at risk on day 5 and nine remaining by day 30. The 30-day survival analysis using Kaplan-Meier curves (Figure 2) showed no statistically significant differences between groups (log-rank test: χ² = 6.398, df = 4, *p* = 0.171). 

Responses according to the administration strategy 

Hemodynamic parameters (MAP), CCI, VIS, and laboratory values (lactate) were analyzed across MB administration strategies (bolus, continuous infusion, bolus + infusion) using χ² tests for categorical outcomes and ANOVA/Kruskal-Wallis for continuous variables (Table 4). 

**Table 4:** Comparative proportions of response, hemodynamic markers, and outcomes by methylene blue administration strategy Values are expressed as n/N (%). Group comparisons were performed with the Pearson χ² test and are reported as χ²(df, N) with the corresponding p value. Statistical significance was set at *p* < 0.05. Criteria and thresholds for the dichotomized variables are detailed in the Methods. Denominators (N) vary by row due to variable-specific missing data. Abbreviations: MAP, mean arterial pressure; VIS, vasoactive inotropic score; CCI, Charlson Comorbidity Index

Variable	Bolus n/N (%)	Infusion n/N (%)	Bolus + infusion n/N (%)	Test statistic, χ² (df, N)	*p*-value
Mortality	27/35 (77.1%)	40/53 (75.5%)	8/10 (80.0%)	0.107 (2, 98)	0.948
Therapeutic response	29/35 (82.9%)	33/53 (62.3%)	9/10 (90%)	6.197 (2, 98)	0.045
MAP	15/34 (44.1%)	24/53 (45.3%)	6/9 (66.7%)	1.573 (2, 96)	0.464
VIS	16/21 (76.2%)	16/29 (55.2%)	7/7 (100.0%	6.173 (2, 57)	0.045
CCI	28/35 (80.0%)	46/53 (86.8%)	8/10 (80.0%)	0.822 (2, 98)	0.670
Lactate	23/35 (67.6%)	28/48 (58.3%)	9/9 (100.0%)	5.929 (2, 91)	0.052

A statistically significant difference in response rate was observed-bolus 29/35 (82.9%), bolus + infusion 9/10 (90.0%), and infusion 33/53 (62.3%) (χ²(2, *N* = 98) = 6.197, *p *= 0.045). No significant differences were found for adverse clinical outcomes (mortality: χ²(2, *N* = 98) = 0.107,* p* = 0.948). VIS and lactate showed favorable trends in the bolus + infusion group (VIS: 7/7 (100%), χ²(2, *N* = 57) = 6.173, *p* = 0.046; lactate: 9/9 (100%), χ²(2, *N* = 91) = 5.929, *p* = 0.052). MAP and CCI did not differ across strategies (MAP: χ²(2, *N* = 96) = 1.573, *p* = 0.455; CCI: χ²(2, *N *= 98) = 0.822, *p* = 0.663). 

Response to MB

Using the Wilcoxon signed-rank test, the MAP increased from 70.5 ± 19.3 to 74.2 ± 22.0 (median 70.8 (56.3-83.0) to 76.2 (65.3-86.4); W = 1413.0; *p* < 0.001) (Table 5). VIS increased from 15.9 ± 34.2 to 92.6 ± 414.6, but with wide dispersion; the median shifted from 6.0 (0.0-15.0) to 21.0 (8.1-68.2) (W = 574.0; *p* < 0.001). The Shock Index decreased from 1.16 ± 0.58 to 1.03 ± 0.47 (median 1.01 (0.75-1.37) to 0.91 (0.72-1.18); W = 1483.0; *p* = 0.003). Creatinine clearance decreased from 41.9 ± 25.7 to 32.3 ± 21.4 (median 38.0 (21.3-55.8) to 26.0 (17.3-43.0); W = 260.5; *p *= 0.002). Lactate showed no significant change (mean 5.5 ± 6.2 to 6.6 ± 7.1; median 1.8 (0.9-10.5) to 2.2 (0.9-15.5); W = 16.0; *p* = 0.130). 

**Table 5:** Pre- and post-treatment changes in physiological variables following methylene blue administration assessed using the Wilcoxon test. Values are mean ± SD at baseline (pre-MB) and after treatment (post-MB). Paired comparisons used the Wilcoxon signed-rank test (two-sided) and are reported as the standardized Z statistic with the corresponding p value. Statistical significance was set at *p* < 0.05. Sample sizes (*n*) reflect pairwise complete cases and therefore vary by variable. Absolute and relative percentage changes from baseline were calculated for mean arterial pressure (MAP), vasoactive-inotropic score (VIS), and lactate levels to better characterize the hemodynamic response after methylene blue administration. Abbreviations: MAP, mean arterial pressure; VIS, vasoactive inotropic score; SI, shock index; ClCr, creatinine clearance

Parameter	Pre-MB (mean ± SD, *n*)	Post-MB (mean ± SD, *n*)	Z	*p*-value
MAP	70.50 ± 19.30 (97)	74.16 ± 21.95 (96)	−3.342	<0.001
VIS	15.92 ± 34.21 (98)	92.57 ± 414.69 (98)	6.567	<0.001
SI	1.16 ± 0.58 (98)	1.03 ± 0.47 (95)	2.958	0.003
ClCr	41.95 ± 25.65 (81)	32. 34 ± 21.44 (55)	3.059	0.002
Lactate	5.53 ± 6.21 (29)	6.56 ± 7.06 (22)	−1.511	0.142

Because clinical response was defined as a composite endpoint (≥10% improvement in MAP, ≥10% reduction in VIS, or ≥10% decrease in lactate), many patients were classified as responders through MAP improvement, even though the cohort as a whole exhibited higher post-treatment VIS. This reflects heterogeneity: while some patients benefited hemodynamically, others required escalation of vasoactive support, producing outliers that increased the mean VIS. Reporting both means and medians helps clarify this divergence. 

Predictors of response and mortality 

As outlined in Table 6, univariate logistic regression identified that a lower pre-treatment VIS was associated with higher odds of response (OR = 0.981 per unit, 95% CI 0.967-0.998; Wald z = −2.247, *p* = 0.025; model LR χ²(1, N = 98) = 7.402,* p* = 0.007). Norepinephrine use before MB was inversely associated (OR = 0.183, 95% CI 0.039-0.869; z = −2.138, p = 0.033), as was a greater ΔNE after MB (OR = 0.070, 95% CI 0.013-0.377; z = −3.093, *p* = 0.002). Compared with bolus, infusion was associated with lower odds of response (OR = 0.34, 95% CI 0.12-0.97; z = −2.026, *p* = 0.043; LR χ²(2, N = 98) = 6.554, *p* = 0.038), whereas bolus + infusion did not differ from bolus (OR = 1.86, 95% CI 0.20-17.58; *p* = 0.587). 

**Table 6:** Univariate logistic regression for predictors of clinical response and mortality. Odds ratios (OR) with 95% confidence intervals (CI); p values from the Wald test (reported with Wald z). Outcome coding: MB Response = 1 (response); Outcome = 1 (event). The reference category for dosing strategy is Bolus. *N* = 98 (varies by predictor due to missing data). Absolute and relative percentage changes from baseline were also calculated for selected hemodynamic variables (e.g., MAP, VIS, lactate) to complement subgroup comparisons. Statistical significance was set at *p *< 0.05. Abbreviations: NE, norepinephrine; ΔNE, change in norepinephrine; MAP, mean arterial pressure; SI, shock index; CCI, Charlson Comorbidity Index

Variable N = 98	MB Response			Outcome		
	OR (95% CI)	Wald z	*p* value	OR (95% CI)	Wald z	*p* value
Comorbidities						
CCI	1.058 (0.810, 1.383)	0.41	0.68	1.28 (0.95–1.71)	1.64	0.101
Laboratory values before MB administration						
Lac	1.052 (0.908 – 1.219)	0.68	0.46	1.060 (0.913–1.231)	0.77	0.444
Vasopressors/Inotropes prior to MB administration						
VIS	0.981 (0.967–0.998)	-2.25	0.03	1.026 (0.986–1.067)	1.27	0.205
NE	0.183 (0.039–0.869)	-2.14	0.03	12.004 (0.198–727.242)	1.19	0.235
Dopamine	0.800 (0.510–1.271)	-1.72	0.09	1.02 (0.88-1.19)	0.24	0.808
ΔNE	0.070 (0.013–0.377)	-3.09	0.00	0.600 (0.239–1.508)	-1.09	0.277
Hemodynamic variables						
MAP	0.995 (0.972–1.019)	-0.39	0.70	0.952 (0.924–0.981)	-3.18	0.001
HR	1.001 (0.986–1.016)	0.16	0.87	0.996 (0.981–1.010)	-0.57	0.568
SI	1.208 (0.540–2.704)	0.46	0.65	2.258 (0.793–6.423)	1.53	0.127
Dosing strategy						
Infusion	0.34 (0.12–0.97)	-2.03	0.04	0.91 (0.33–2.50)	-0.18	0.857
Bolus + infusion	1.86 (0.20–17.58)	0.54	0.59	1.19 (0.21–6.75)	0.19	0.848

For adverse clinical outcomes, only post-treatment MAP showed a significant association (OR = 0.952 per mmHg, 95% CI 0.924-0.981; Wald z = −3.181, *p* = 0.001). Other predictors, including VIS, lactate, pH, ΔNE, dosing strategy, norepinephrine, and other hemodynamic variables, were not associated (all* p* > 0.05). Consistent with this, adding a dosing strategy did not improve model fit for this endpoint (LR χ²(2, N = 98) = 0.110, p = 0.947). 

In the multivariable logistic regression for clinical response (complete cases *N* = 80), a lower pre-treatment VIS remained independently associated with higher odds of response (aOR = 0.979, 95% CI 0.963-0.996; Wald z = −2.413, *p* = 0.016), whereas MAP, shock index, creatinine clearance, and Charlson index were not significant (all *p* > 0.05) (Table 7). Model fit was modest (LR χ²(5, *N* = 80) = 8.018, *p* = 0.155; Nagelkerke R² = 0.136). 

**Table 7:** Multivariate model for prediction of clinical response and adverse clinical outcome Multivariable logistic regression with Wald tests for coefficients. Values are adjusted odds ratios (aOR) with 95% CI, reported with the corresponding Wald z and p value. Models were estimated on complete cases (N = 80). Outcome coding: Response = 1; Adverse outcome = 1. Reference category: dosing strategy = Bolus. Model fit: Response—LR χ²(5, N = 80)=8.018, *p* = 0.155; Nagelkerke R²=0.136. Adverse outcome—LR χ²(7, N=80)=15.692,* p* = 0.028; Nagelkerke R² = 0.272. Statistical significance was set at *p* < 0.05. Abbreviations: MB, methylene blue; MAP, mean arterial pressure; SI, shock index; ClCr, creatinine clearance; VIS, vasoactive inotropic score; CCI, Charlson Comorbidity Index

Patient variables related to MB administration	MB response			Outcome		
	OR (95% CI)	Wald z	*p* value	OR (95% CI)	Wald z	*p* value
CCI	0.942 (0.187–4.759)	-0.72	0.942	1.457 (0.250–8.487)	0.418	0.676
Hemodynamic variables						
MAP	0.983 (0.948–1.020)	-0.905	0.365	0.933 (0.889–0.979)	-2.797	0.005
SI	1.148 (0.333–3.951)	0.218	0.827	0.442 (0.099–1.980)	-1.068	0.286
ClCr	1.006 (0.984–1.028)	0.568	0.570	1.014 (1.030–1.866)	-0.227	0.821
VIS	0.979 (0.963–0.996)	-2.413	0.016	1.018 (0.975–1.063)	0.829	0.407
Dosing strategy						
Bolus	----------		----------	0.930 (0.095–9.080)	-0.62	0.950
Infusion	----------		----------	0.933 (0.251–3.467)	-0.103	0.918

For adverse clinical outcomes, a higher pre-treatment MAP was associated with lower odds of the event (aOR = 0.933 per mmHg, 95% CI 0.891-0.979; Wald z = −2.797, *p *= 0.005), while VIS, Charlson index, creatinine clearance, and shock index were not significant. The dosing strategy also showed no association (infusion vs. bolus: aOR = 0.93, 95% CI 0.25-3.47; z = −0.103; p = 0.918; bolus + infusion vs. bolus: aOR = 0.93, 95% CI 0.10-9.08; z = −0.062; *p* = 0.950). Model fit for this endpoint was acceptable (LR χ²(7, N = 80) = 15.692, *p* = 0.028; Nagelkerke R² = 0.272). 

## Discussion

The findings of this study support the use of MB as an adjuvant therapy in patients with refractory shock, especially in those with a lower initial vasopressor requirement and who achieve a sustained increase in the MAP after the intervention [4,14,15]. Bolus administration, alone or followed by continuous infusion, may represent an effective scheme from a pharmacodynamic perspective. Similarly, changes in norepinephrine dose and the VIS could be used as dynamic markers to guide therapeutic decisions and assess early response. However, the impact of MB appears to depend more on the patient’s physiological response and clinical status at the time of intervention than on baseline characteristics or the dosing regimen used. 

Although the VIS increased at the population level, this reflects the heterogeneity of clinical response: while some patients improved hemodynamically and required less vasopressor support, others deteriorated, resulting in extreme values that skewed the mean VIS. Median values and subgroup trends provide a more accurate reflection of response.

From a pathophysiological standpoint, the effectiveness of MB in refractory shock lies in its ability to counteract vasoplegia induced by excessive nitric oxide (NO) production. In states such as septic shock, activation of inducible nitric oxide synthase (iNOS), and stimulation of soluble guanylate cyclase (sGC) lead to increased cyclic GMP (cGMP), perpetuating vasodilation [16,17]. MB inhibits both iNOS and sGC, thereby reducing cGMP levels and reversing refractory vasodilation [7,9]. This mechanism explains its vasoconstrictive effect, with potential to improve MAP and restore tissue perfusion in the early phases of shock, particularly septic shock, when iNOS and sGC activity is most pronounced [18,19]. Current recommendations advocate for the combined use of multiple therapeutic agents to restore perfusion and minimize adverse effects associated with monotherapy [8,20]. 

In this retrospective study, a predominance of male patients was observed (65.3%, n = 64). The average hospital stay was 13.38 ± 16.31 days, while the mean ICU stay was 11.15 ± 15.15 days. Regarding clinical outcomes, 76.5% (n = 75) of patients died; moreover, 84.68% (n = 83) of these deaths occurred within the first 30 days. This high mortality rate may be explained by several clinical and methodological factors. Most patients had a high comorbidity burden, with nearly 70% (n = 68) presenting a severe CCI. 

In addition, mortality in patients with shock varies depending on the underlying etiology and severity. Cardiogenic shock is associated with the highest mortality, ranging from 43.2% to 70%, while 30-day mortality in septic shock ranges from 34% to 40% [21-23]. Although these findings suggest an elevated mortality rate, it was not possible to calculate standardized prognostic scores due to insufficient data in the medical records. Furthermore, the absence of a control group without MB limits the ability to attribute mortality to the treatment or to the natural progression of the disease. 

MB was primarily used in septic shock cases, supporting its potential use in this population according to previous literature [14,15]. The burden of comorbidities was high, as evidenced by the CCI, highlighting the complexity of clinical management. The most frequent conditions included cardiovascular (69.4%, n = 68), central nervous system (52.0%, n = 51), respiratory (27.5%, n = 27), and oncologic (18.4%, n = 18) diseases. The CCI is a key predictor of patient prognosis regardless of diagnosis, with higher scores being associated with increased short-term mortality [24]. Finally, when applying the clinical response criteria defined in the methodology, 73.47% (n = 72) of patients showed an initial favorable response to MB treatment. 

International guidelines for managing septic and cardiogenic shock recommend a stepwise strategy based on pathophysiology and tailored to clinical response. Initial fluid resuscitation with crystalloids is emphasized, followed by norepinephrine as the first-line vasopressor and inotropic support in persistent myocardial dysfunction [25,26]. In this study, initial management included fluid replacement and vasopressor/inotropic support, aligning with current recommendations, where norepinephrine (91.8%, n = 90) was the vasopressor of choice. The high use of albumin (91.8%, n = 90) and saline solution (85.7%, n = 84) also reflects common volume resuscitation practices [25]. The use of dobutamine in 22.4% (n = 22) of cases suggests a frequent need for additional inotropic support, possibly in the setting of concurrent myocardial dysfunction. 

Currently, several studies assess overall or 30-day mortality, but many do not stratify outcomes by shock etiology [27]. In our cohort, 30-day survival analysis by shock type did not show statistically significant differences (χ² = 6.398, *p* = 0.171), although relevant clinical trends were noted. Cardiogenic shock had the lowest median survival (2 days) with high early mortality, consistent with rapid progression due to cardiac output failure [28]. Mixed shock reached 100% mortality (n = 5) before day 30; however, this should be interpreted with caution, given the small sample and pathophysiological complexity. In contrast, distributive shock (including septic shock) showed better performance, with higher median and absolute 30-day survival, suggesting a favorable therapeutic response, timely management, or a greater proportion of reversible conditions [29].

Although mortality did not differ significantly among shock types, patients with distributive and septic shock demonstrated a trend toward better hemodynamic response following MB administration. This observation suggests that MB may be more effective in vasoplegic phenotypes characterized by excessive nitric oxide-mediated vasodilation.

Several parameters were evaluated before and after MB administration. A significant improvement in MAP (from 70.5 ± 19.3 to 74.2 ± 21.9 mmHg;* p *< 0.001) and a reduction in SI (from 1.16 ± 0.58 to 1.03 ± 0.47; *p *= 0.002) were observed, reflecting a favorable hemodynamic response [12,13]. However, a decrease in creatinine clearance (from 41.9 ± 25.6 to 32.3 ± 21.4;* p* = 0.002) was also documented, possibly indicating persistent renal hypoperfusion or acute tubular injury due to clinical severity.

An important nuance concerns the behavior of VIS. At the cohort level, VIS increased significantly after MB (p < 0.001). Given the composite definition of clinical response (≥10% improvement in MAP, ≥10% reduction in VIS, or ≥10% decrease in lactate), many patients were classified as responders through MAP improvement despite a population-level increase in VIS. Moreover, VIS exhibited a highly skewed distribution with extreme values; therefore, reporting medians and IQR better captures central tendency (from 6.0 (0.0-15.0) to 21.0 (8.1-68.2)). This pattern is compatible with heterogeneity in response: a subgroup benefited hemodynamically, while another required escalation of vasoactive support, inflating mean VIS. This underscores the need to evaluate combined markers (hemodynamic and metabolic) to assess treatment response [15,30,31]. 

Multiple meta-analyses and randomized clinical trials support MB’s role in shock management, particularly in distributive shock. These studies consistently report reductions in hospital stay, ICU days, and mechanical ventilation duration; however, evidence regarding its impact on mortality remains limited and heterogeneous. Our findings are broadly consistent with these data, although differences in study design, populations, and therapeutic protocols complicate direct comparisons [20,30,32-34]. 

In univariate analysis, higher pre-treatment VIS was associated with lower probability of clinical response (OR: 0.981; p = 0.025), indicating that greater vasopressor requirements correlate with reduced effectiveness. A larger reduction in norepinephrine dose after MB (ΔNE) was associated with a higher response (OR: 0.070; p = 0.002). In the multivariable model, only pre-treatment VIS remained an independent predictor (OR: 0.979; p = 0.016), suggesting that greater vasoactive support at baseline is linked to lower efficacy and possibly reflects more advanced hemodynamic compromise. Other variables, including CCI, lactate, MAP, and administration strategy, were not statistically significant in either model [9,32,33]. 

Regarding clinical outcomes, both univariate and multivariable analyses showed that a higher post-treatment MAP was associated with a lower risk of adverse outcome (OR ≈ 0.93 per mmHg; p = 0.005) [4,14,15]. Current evidence suggests that MAP should be maintained above 65 mmHg to ensure organ perfusion and improve survival in shock patients [25,35]. Other variables (CCI, SI, ClCr, and administration mode) showed clinically relevant trends but were not significantly associated with outcomes (p > 0.05), likely due to limited sample size and data heterogeneity, reducing model power. Future studies with larger samples could clarify whether these variables may serve as independent predictors. 

Finally, the dosing strategy influenced clinical efficacy. A statistically significant difference in response rates was observed, with bolus 82.9% (29/35), bolus plus infusion 90.0% (9/10), and infusion 62.3% (33/53) (*p* = 0.045). These findings support the pharmacodynamic rationale for achieving a rapid plasma peak with an initial bolus, alone or followed by infusion, to promptly restore vascular tone and perfusion. This benefit may be particularly relevant in early stages of vasoplegia when NO/sGC-mediated mechanisms are dominant. While evidence on combination regimens remains limited, prospective studies are needed to confirm efficacy. An initial bolus may allow rapid attainment of effective plasma concentrations for immediate effect, whereas a subsequent infusion could help maintain therapeutic exposure over time [27,36]. 

MB may be integrated into a multimodal strategy for refractory shock, particularly in catecholamine-resistant states. Randomized trials and meta-analyses suggest that early administration can reduce vasopressor requirements and hospital length of stay [18,30]. Its potential to improve MAP and reduce catecholamine exposure justifies consideration in local clinical algorithms. Controlled, multicenter studies incorporating prognostic tools (e.g., SOFA and APACHE II) and biomarkers are needed to delineate the patient profile most likely to benefit. 

This study has several limitations inherent to its retrospective design. The lack of a control group limits causal inference, as the indication, timing, and dosing of MB were not standardized and depended on clinical judgment, introducing selection bias and heterogeneity. In addition, complementary variables such as inflammatory biomarkers, fluid balance, and mechanical ventilation parameters were not consistently recorded. The classification of shock type was based on clinical records rather than confirmation through invasive hemodynamic monitoring. Standardized severity scores such as SOFA or APACHE II were not consistently available in the medical records, which limited the ability to correlate illness severity with mortality or treatment response. A comparable control group of refractory shock patients who did not receive MB could not be established due to incomplete information on vasoactive dosing and timing. Future multicenter registries may enable such comparative analyses.

Although conducted at a single institution, the ICU involved operates with resources and clinical capabilities similar to tertiary-level international centers, which supports partial extrapolation of results to comparable settings. Nevertheless, as this study was carried out in a private tertiary hospital in Costa Rica, variations in healthcare infrastructure, patient population, and clinical protocols may limit the external validity and generalizability of these findings to other contexts.

## Conclusions

This retrospective study supports the use of MB as an adjunctive therapy for refractory shock, particularly in patients with lower initial vasopressor requirements and those showing an increase in the MAP after treatment. The MAP and VIS functioned as dynamic markers of response, although the population-level increase in VIS reflected heterogeneous outcomes, underscoring the need for composite endpoints such as MAP, VIS, and lactate.

Distributive and septic shock demonstrated better outcomes than other subtypes, suggesting a more favorable response in vasoplegic phenotypes. Bolus or bolus-plus-infusion regimens achieved higher response rates than continuous infusion alone.

While hemodynamic improvements were observed, the lack of metabolic changes, such as lactate reduction, highlights the complexity of refractory shock. Future studies should integrate dynamic inflammatory biomarkers and microcirculatory monitoring to strengthen physiological and clinical evidence.

Given the absence of a control group and the non-standardized treatment approach, findings should be interpreted cautiously. Prospective multicenter trials and standardized protocols are needed to confirm these results and guide clinical application in critical care settings.
